# Impact of the new rural social pension insurance on the health of the rural older adult population: based on the China health and retirement longitudinal study

**DOI:** 10.3389/fpubh.2023.1310180

**Published:** 2023-11-14

**Authors:** Yuegang Song, Changqing Song, Ziqi Wang, Guoheng Hu

**Affiliations:** ^1^School of Business, Henan Normal University, Xinxiang, China; ^2^Shengxiang School of Business, Sanda University, Shanghai, China

**Keywords:** new rural social pension insurance, rural older adult population health, logistic regression model, mediating effect, moderating effect

## Abstract

The health issues of China’s older adult population in rural areas have been receiving increasing attention with the continuous expansion of the nation’s ageing population and the continuous promotion of urban–rural integration. The impact of the new rural social pension insurance (NRSPI) on the health of the rural older adult population, the mechanism of its action and how old-age service can be improved and optimised according to the health needs of the rural older adult population are urgent and realistic challenges. Based on survey data from the China Health and Retirement Longitudinal Study in 2015 and 2018, this study applies a multivariate ordered logistic regression model to explore the impact mechanism and effect of the NRSPI on the older adult population health in rural China while controlling for endogeneity. The results show that participation in the NRSPI can significantly improve the health of the rural older adult population at a 1% level. The results of the heterogeneity test reveal that the NRSPI has a significant impact on the self-reported health of the rural older adult at a 1% level, with a significantly positive impact on the mental and physical health of rural female older adult, whereas the impact on male older adult is not significant. The mediating effect test results show that medical services, food access and entertainment activities have a mediating effect on the new rural social endowment insurance. The results of the moderating effect test indicate that the NRSPI regulates 7.8% of the effect of physical health on mental health and 10.7% of the effect of mental health on physical health. Based on these findings, this study proposes to strengthen the construction of healthy lifestyle guidance and emotional support systems while improving the NRSPI’s participation rate and treatment level to meet the diverse health service needs of different older adult groups.

## Introduction

Since the 1990s, China has experienced a sustained rapidly ageing population (1). The scale and proportion of the older adult population are generally on the rise and the rate of growth has been accelerating (2). According to the 2020 National Ageing Development Bulletin issued by the National Health Commission of China and the seventh national census data, the number of older adult people aged 60 and over and 65 and over in China reached 264 million and 190 million, respectively. The proportion of the total population increased from 13.26% and 8.87% during the sixth national census to 18.7% and 13.5%, respectively. More than 50% of the older adult live in rural areas with relatively limited economic and social development, and the degree of ageing in rural areas has been consistently higher than that in cities and towns. Due to imbalanced economic development and inadequate social security benefits, the health service needs of the older adult in rural China are particularly severe (3). The health problems of the rural older adult are reflected in self-reported health as well as actual physical and mental health figures. In addition, the relative poverty caused by the considerable income gap of the rural older adult and health inequality caused by urban–rural differences and uneven distribution of pensions must also be urgently addressed. To improve the health of the older adult in rural areas, the Outline of the 14th Five-Year Plan for China’s National Economic and Social Development, the (14th Five-Year) National Plan for the Development of the Cause of Ageing and the Pension Service System and the 14th Five-Year Plan for Healthy Ageing in 2022 clearly recognised that the health of the older adult must be raised as a national strategy, and a plan to promote the achievement of a comprehensive and continuous health service system for the older adult covering urban and rural areas, rationally allocate health services for the older adult (particularly health service resources for the rural older adult) and continuously improve the health of the rural older adult population.

In the face of China’s increasingly severe ageing trend and the health inequality of the older adult population in urban and rural areas, the traditional family pension model in rural areas has been weakened. Further improving the social pension insurance system is crucial for ensuring the basic lives of the rural older adult and improving the health of the rural older adult population. In this context, the State Council of China began to conduct pilot reform via the new rural social pension insurance (NRSPI) in 2009, basically achieving full coverage in rural areas in 2012. The purpose of the NRSPI is to address the problem of inadequate health services for the rural older adult to achieve a sense of security. As a continuation of China’s livelihood security system, the NRSPI is the core of the existing rural social old-age security system and the key to ensuring the equalisation of urban and rural old-age services. Its biggest characteristic is to adopt the mode of combining three financing channels of individual payment, collective subsidy and government subsidy. Through the external subsidy support of the government, it provides a new pension income for the rural older adult to alleviate their living pressure, improve their living standards, and bring some support and guarantee to their economic life. Accordingly, investigations of the socio–economic effects of the NRSPI have also attracted academic attention. As the basic insurance system for the older adult in rural China, can the NRSPI achieve the goal of improving the health of the rural older adult population? If so, through what channels does the NRSPI effectively advance health protection for the rural older adult? What is its mechanism of action? These questions require investigation. Based on China’s population ageing, the goal of healthy ageing and the policy orientation of the Healthy China initiative, this study use the multivariate ordinal logistic regression model, narrows the traditional research scope to the impact of the NRSPI on the health status of rural older adult population by constructing the mediating effect and moderating effect hypothesis to propose corresponding policy implications regarding the optimal path for pension service in the current NRSPI system.

## Literature review

Since the end of the 20th century, an increasing number of nations have experienced rapidly ageing societies. The issues of pension insurance and older adult population health have become urgent challenges for many governments. As the academic community has increasingly examined health inequality among older adult populations, the impact of China’s NRSPI on the health of the rural older adult population has become a popular topic of academic research. Based on previous research, this study primarily presents three topics in the relevant literature, including the effects of NRSPI policy implementation, the influencing factors of older adult population health and the impact of the NRSPI on older adult population health.

### Research on the effect of NRSPI implementation and its impact

In previous research, although the NRSPI was merged with urban residents’ basic pension insurance in 2014, the academic community is still accustomed to referring to it as the NRSPI. The NRSPI is significant aspect of China’s social security system reform and development and a crucial measure to protect the livelihoods and welfare of the older adult in rural areas. As the research object of interdisciplinary fields such as economics, politics, demography and sociology, the challenge of rural old-age care has been a popular research topic. Since NRSPI policy implementation, previous research has conducted a series of studies on the conditions, methods and effects of insurance participation (4). These studies have focused on analysing the advantages and disadvantages of NRSPI implementation (5–8), and the majority of the research has focused on the impact of the NRSPI on the economic status of rural older adult (9). Some studies have analysed the effect of the NRSPI on older adult population income and poverty reduction, consumption promotion and crowding out effects of savings from an economic perspective (10–14). Some studies have also explored the impact of the NRSPI on the family lives of the rural older adult, its impact on the traditional family pension model and its impact on grandchild care from the perspective of inter-generational support (15–20). Some studies have examined the impact of the NRSPI on the labour supply of the rural older adult, including agricultural labour supply and other labour supply related to land transfer (21–24).

### Research on the influencing factors of older adult population health

With growing emphasis on investigating older adult population health in the academic community, based on Grossman’s health needs theory and associated model (25), considerable research has examined the health of the older adult population from multiple fields and perspectives, exploring a variety of factors, including individual characteristics, urban–rural differences, socio–economic status and social security (26–33). In addition, based on the factors affecting health inequality proposed by Fleurbaey and Schokkaert (34, 35), combined with the environmental factors of China’s unique urban and rural household registration system, some studies have focused on the health inequality of the rural older adult population (36–42).

### Research on the impact of NRSPI implementation on older adult population health

In recent years, with the implementation of China’s policy to promote basic old-age care services for all older adult citizens, the academic community has also applied a variety of data resources and conducted extensive and in-depth research on the impact mechanism between the NRSPI and the challenges of addressing older adult population health from multiple perspectives. Some research has examined whether the NRSPI policy has promoted older adult population health, including physical and psychological depression and subjective well-being dimensions (43–47). In terms of research methods, some scholars have explored the impact mechanism of the NRSPI on older adult population health based on micro-sectional data using propensity score matching (PSM) or instrumental variable approaches. Some studies have constructed balanced panel data using the PSM difference-in-differences (PSM-DID) method to evaluate NRSPI impacts. These studies have primarily empirically tested the impact of the NRSPI on income, consumption, labour supply, inter-generational support, poverty reduction and other aspects of its impact on the older adult (48–51).

### Related literature review

The literature review reveals that previous research has generally investigated the correlation between pension insurance and older adult population health, conducting research on the performance of pension insurance on older adult population health from multiple perspectives; however, some considerations need to be deepened and improved. First, the existing research objects primarily include the entire older adult population, and a limited number of studies have conducted research on the rural older adult population based on China’s unique urban and rural household registration. In addition, previous research on older adult population health has been largely based on self-reported or physical health, and few scholars have included mental health considerations. Second, previous literature has rarely explored the path through which the NRSPI system promotes rural older adult health, lacking considerations of the impact path of the NRSPI system and mediating effect analyses. It is challenging to accurately quantify how the NRSPI system has improved the health of the rural older adult. Third, primary research conclusions regarding the impact of the NRSPI system on the health of the older adult are contradictory. This could be related to the year(s) of the survey data examined. The time span of the cross-sectional data examined in the previous research has been earlier, and the span of the balanced panel data constructed has been shorter, without consideration of the inherent hysteresis of the NRSPI’s institutional effects.

Based on the above, this study uses data from the most recent China Health and Retirement Longitudinal Study (CHARLS) which were conducted in 2015 and 2018, takes the rural older adult population in China as the main research object, using self-reported health, mental health and physical health as explanatory variables to evaluate the health of the older adult and applies health demand, life cycle and social support theories as the primary theoretical basis. Using a multivariate ordered logistic model, this study examines the effects of NRSPI implementation on the health status of the older adult in rural China and its heterogeneous effects on rural older adult of different genders. The study also proposes mediating and moderating effect hypotheses to analyse and test the effect of the NRSPI on rural older adult population health, providing scientific and feasible theoretical insights for China to further improve the basic rural pension insurance system and improve rural older adult health.

### Marginal contributions

Referencing previous research ideas, this study aims to comprehensively explore the impact of the NRSPI on China’s rural older adult population health, with the following four contributions. First, in terms of research objects, this study takes the rural older adult divided by China’s unique urban and rural household registration as the research object, classifies older adult health into self-rated, mental and physical health and empirically analyses the impact of the NRSPI on the health of the rural older adult to provide a valuable policy reference for improving China’s NRSPI system and rural older adult health. Second, in terms of research methods, this study applies the multivariate ordinal logistic regression model to the latest CHARLS data in 2015 and 2018 to analyse the impact of the NRSPI on rural older adult population health. The research conclusions provide valuable insights for improving the implementation of this new rural insurance policy. Third, in terms of theoretical mechanisms, this study constructs a theoretical model to quantify the effects of the NRSPI on rural older adult health and proposes research hypotheses regarding mediating and moderating effects to deepen the understanding of the relationship between the NRSPI and rural older adult health and investigates how the NRSPI has improved medical service, leisure and entertainment activities and nutrition for China’s rural older adult.

## Theoretical analysis and research hypotheses

This study reference the research of Wu et al. (52), through theoretical analysis, explain how the implementation of NRSPI will affect the health of the older adult population from the theoretical logic, and puts forward three propositions as the hypothesis of this empirical research.

Based on the health demand theory of Grossman (25), this study includes age, gender, marital status, income level, recreational activities, social class and social insurance as potential influencing factors. This study argues that health can be considered as an investment product in which income and recreational activities can increase individuals’ health stock. Individuals can protect their health by procuring medical services and spending leisure and entertainment time. As a social insurance factor in the health demand function, the NRSPI increases older adult pension income and leisure and entertainment time, which subsequently improves rural older adult health (52). Specifically, income is a significant factor in determining the health of the older adult in rural areas. Pension income can improve individuals’ socio–economic status, which allows the older adult to have sufficient risk resistance, less mental stress and higher self-reported health when facing health risks (53). Pension income can also improve health awareness and mental health, cause the older adult to prioritise their own health, provide more choices in medical treatment and positively impact physical health (43). In addition, when the rural older adult secure a stable pension income, leisure and entertainment activities are expected to increase, which also improves rural older adult health. Therefore, this study proposes Hypothesis 1.

*Hypothesis 1:* The NRSPI has a positive impact on rural older adult population health.

Referencing the life cycle theory of Modigliani (54), this study argues that rational economic individuals will allocate income and make decisions regarding consumption and savings across the entire life cycle according to the principle of utility maximisation. As a stable source of income, the NRSPI has impacted the medical consumption decisions of the rural older adult throughout the life cycle, increasing consumption expenditure for disease prevention (55), reducing the occurrence of chronic diseases, promoting the use of medical services and improving the activities of daily life, which significantly enhances the physical health of the insured older adult (56, 57). The NRSPI has also increased rural older adult families’ disposable income, reduced the uncertainty of future income and inhibited current pension-saving practices, with a significant effect on healthy consumption and nutritious dietary consumption (58). The policy has increased the variety of food intake and promoted improved eating habits and dietary balance, ultimately enhancing the health performance and physical health status of the rural insured older adult (43). Therefore, this study proposes Hypothesis 2.

*Hypothesis 2:* The NRSPI has a positive impact on the physical health of the older adult in rural areas by increasing medical services through health care disbursement and diversifying the variety of food intake to promote a balanced diet.

Based on the theory of social support, this study contends that when the economic support of the children of the older adult in rural areas is inadequate to support the older adult (26), support at the macro level of society can alleviate the economic pressure experienced by the older adult. Therefore, as a formal social support provided by the government, the NRSPI delivers stable pension income for the older adult in rural areas, replacing labour income, reducing labour time, increasing disposable leisure time to participate in more leisure and other recreational activities, alleviating concerns regarding old age, improving life satisfaction and boosting mental health and subjective well-being (44). Increased recreational activities also improve the cognitive functions of the older adult, generating confidence in the future, increasing happiness and satisfaction and more optimism regarding personal health evaluation, which improves the level of self-reported health (47, 59). Therefore, this study proposes Hypothesis 3.

**Figure 1:**
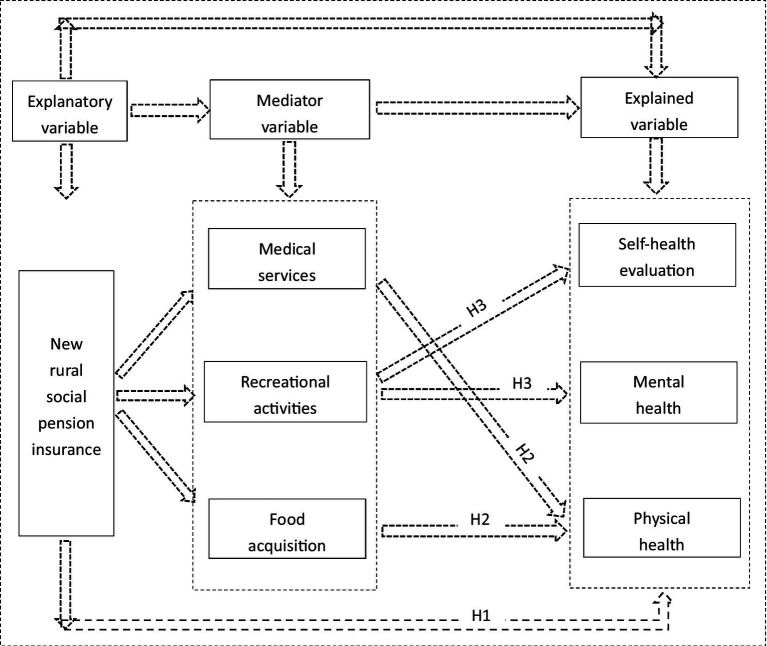
The research roadmap.

*Hypothesis 3:* The NRSPI improves the mental and self-reported health of the rural older adult by increasing opportunities for recreational activities (Figure 1).

## Empirical analysis

### Model setting

#### Benchmark regression model

Based on the above theoretical analysis, to examine the impact of the NRSPI on rural older adult population health, this study refers to Werner et al. (60), and uses the health level of the rural older adult (self-reported health evaluation, mental health and physical health) as the explained variable, and whether the NRSPI was implemented as the explanatory variable. The mediating variables are also incorporated and other variables that may affect health are introduced as control variables, and the multivariate ordered logistic model is used as the benchmark regression model. The details are as follows:


(1) $$sfe_{\mathrm{it}}=\alpha_{0}+\alpha_{1}\mathrm{nrsp}i_{\mathrm{it}}+\alpha_{2}\mathrm{control}s_{\mathrm{it}}+\psi_{i}+\psi_{t}+\varepsilon_{\mathrm{it}}$$

(2) $$mth_{\mathrm{it}}=\alpha_{3}+\alpha_{4}\mathrm{nrsp}i_{\mathrm{it}}+\alpha_{5}\mathrm{control}s_{\mathrm{it}}+\psi_{i}+\psi_{t}+\varepsilon_{\mathrm{it}}$$

(3) $$\mathrm{ps}h_{\mathrm{it}}=\alpha_{6}+\alpha_{7}\mathrm{nrsp}i_{\mathrm{it}}+\alpha_{8}\mathrm{control}s_{\mathrm{it}}+\psi_{i}+\psi_{t}+\varepsilon_{\mathrm{it}}$$

where subscripts i and t represent the rural older adult individual and year, respectively; $sfe_{\mathrm{it}}$ , $mth_{\mathrm{it}}$ and $\mathrm{ps}h_{\mathrm{it}}$ represent the self-reported health evaluation, mental and physical health of individual i in year t ; $\mathrm{nrsp}i_{\mathrm{it}}$ represents whether individual i participated in the NRSPI in year t ; $\psi_{i}$ and $\psi_{t}$ represent individual and year fixed effects, respectively; $\mathrm{control}s_{\mathrm{it}}$ is a series of related control variables at the individual level; and $\varepsilon_{\mathrm{it}}$ represents the random disturbance term.

#### Mediating mechanism test model

In addition to the direct effects embodied in Eqs 1–3, to explore possible indirect effects, this study references Wen et al. (61), testing the mediating effects of medical services, food acquisition and recreational activities between the NRSPI and rural older adult population health. The specific form of the model is set as follows:


(4) $$\mathrm{lnMi}d_{\mathrm{it}}=\beta_{0}+\beta_{1}\mathrm{nrsp}i_{\mathrm{it}}+\beta_{2}\mathrm{control}s_{\mathrm{it}}+\psi_{i}+\psi_{t}+\varepsilon_{\mathrm{it}}$$

(5) $$sfe_{\mathrm{it}}=\eta_{0}+\eta_{1}\mathrm{nrsp}i_{\mathrm{it}}+\eta_{2}\mathrm{lnMi}d_{\mathrm{it}}+\eta_{3}\mathrm{control}s_{\mathrm{it}}+\psi_{i}+\psi_{t}+\varepsilon_{\mathrm{it}}$$

(6) $$mth_{\mathrm{it}}=\eta_{4}+\eta_{5}\mathrm{nrsp}i_{\mathrm{it}}+\eta_{6}\mathrm{lnMi}d_{\mathrm{it}}+\eta_{7}\mathrm{control}s_{\mathrm{it}}+\psi_{i}+\psi_{t}+\varepsilon_{\mathrm{it}}$$

(7) $$\mathrm{ps}h_{\mathrm{it}}=\eta_{8}+\eta_{9}\mathrm{nrsp}i_{\mathrm{it}}+\eta_{10}\mathrm{lnMi}d_{\mathrm{it}}+\eta_{11}\mathrm{control}s_{\mathrm{it}}+\psi_{i}+\psi_{t}+\varepsilon_{\mathrm{it}}$$

Equations 4–7 examine the mediating effect of $\ln Mid_{\mathrm{it}}$ using a step-wise regression. The potential mediating variables include medical services ( msv ), food acquisition ( fas ) and recreational activities ( rac ), and the remaining variables are the same as above.

#### Moderating effect test model

Referencing Jiang (62), this study constructs the following moderating effect model:


(8) $$\begin{array}{l} mth_{\mathrm{it}}=\sigma_{0}+\sigma_{1}\mathrm{nrsp}i_{\mathrm{it}}+\sigma_{2}mth_{\mathrm{it}}\times \mathrm{ps}h_{\mathrm{it}}+\sigma_{3}\mathrm{ps}h_{\mathrm{it}}\\ +\sigma_{4}\mathrm{control}s_{\mathrm{it}}+\psi_{i}+\psi_{t}+\varepsilon_{\mathrm{it}} \end{array}$$

(9) $$\begin{array}{l} \mathrm{ps}h_{\mathrm{it}}=\sigma_{5}+\sigma_{6}\mathrm{nrsp}i_{\mathrm{it}}+\sigma_{7}mth_{\mathrm{it}}\times \mathrm{ps}h_{\mathrm{it}}\\ +\sigma_{8}mth_{\mathrm{it}}+\sigma_{9}\mathrm{control}s_{\mathrm{it}}+\psi_{i}+\psi_{t}+\varepsilon_{\mathrm{it}} \end{array}$$

The results of the moderating effect test primarily concern the significance of the interaction term coefficients $\sigma_{2}$ and $\sigma_{7}$ to verify the positive and negative and significance of the product coefficients of mental and physical health in Eqs 8, 9. The remaining variables are the same as above.

### Variable descriptions

#### Explanatory variables

The explanatory variable in this study is rural older adult individuals’ participation in the NRSPI, which comes from the question: Do you participate in the new rural insurance? In the CHARLS questionnaire. This is a dummy variable, taking the value of 1 if the respondent participated in the NRSPI, otherwise, a 0 value is assigned.

#### Explained variables

##### Measurement model

This study refers to the practice of Haan and Uhlendorff (63), and prepares five options for the design of the questionnaire on the health status of the rural older adult: very good, good, general, bad, very bad. This is the explanatory variable of this study, covering the possible aspects of the health status of the rural older adult. Self-reported health evaluation, mental health and physical health, which are all ordered categorical variables with values of 1–5, which is a typical sortable choice. Therefore, the multivariate ordered Logistic model is used to estimate this effect, and the basic model is constructed as follows:


(10) $$P\left(y=j|x_{i}\right)=\frac{1}{1+e^{-\left(\alpha +\beta x_{i}\right)}}$$

where $x_{i}$ represents the i index variable, and y is the actual observation value, which is assigned to 1, 2, 3, 4 or 5, respectively, representing the probable older adult individual’s health status in each option. The potential implicit variable $y^{*}$ is introduced into the ordered logistic model as a unidirectionally observable value to examine the health status of the older adult, where $y^{*}$ satisfies the following form:


(11) $$y^{*}=\mathrm{AX}+\varepsilon_{i}$$

where X is the explanatory variable, A is the parameter vector to be estimated and $\varepsilon_{i}$ is the model intercept. γ represents the critical demarcation point of the unknown results of the health status of the middle-aged and older adult people in this study; that is, $\gamma_{1}$ , $\gamma_{2}$ , $\gamma_{3}$ and $\gamma_{4}$ , representing four demarcation points. After obtaining the parameter estimates of $\varepsilon_{i}$ and $\varepsilon_{i}$ , the probability of each value of y can be obtained as follows:


(12) $$P\left(y\leq j|X\right)=\frac{e^{-\left(\alpha +\beta x_{i}\right)}}{1+e^{-\left(\alpha +\beta x_{i}\right)}}$$

#### Measurement method

##### Self-reported health evaluation ( sfe )

This study references the CHARLS question: what do you think of your health? As the measure of self-reported health. The responses include very good, good, general, bad or very bad, and the specific assignments are presented in Table 1.

**Table 1:** Variable definition and descriptive statistics.

Variable name	Index selection	Variable definition	Maximum value	Minimum value	Mean value
Explanatory variable	nrspi	Participation = 1; not participated = 0	1	0	0.761
Explained variable	sfe	Very bad = 1; bad = 2; general = 3; good = 4; very good = 5	5	1	3.092
	mth	Very bad = 1; bad = 2; general = 3; good = 4; very good = 5	5	1	3.587
	psh	Very bad = 1; bad = 2; general = 3; good = 4; very good = 5	5	1	3.255
Control variable	age	The actual age of the older adult	108	60	68.786
	sex	Male = 1; female = 2	2	1	1.531
	mst	With spouse = 1; without spouse = 0	1	0	0.782
	icl	Gross annual income	8922.369	0	6545.492
	sst	1 represents the lowest level; 10 represents the highest level	10	1	4.055
Mediator variable	msv	Sufficient = 1; insufficient = 0	1	0	0.858
	fas	Sufficient = 1; insufficient = 0	1	0	0.446
	rac	Participation = 1; not participate = 0	1	0	0.253

The three digits after the decimal point are rounded.

##### Mental health ( mth )

This study uses life satisfaction, cognitive ability and depression indices to measure the mental health of the rural older adult. To measure mental health, the life satisfaction index references the CHARLS questionnaire’s DC028: are you satisfied with your life, which includes responses of extremely satisfied, very satisfied, relatively satisfied, not very satisfied and not satisfied at all. At the same time, according to the cognitive assessment scale and the cognitive portion of the CHARLS questionnaire’s DC01–DC08 and DC19–DC27 questions, measures the cognitive level of the rural older adult in terms of orientation, reaction and memory. We obtain continuous variables referencing these questions. A higher score indicates higher cognitive abilities. In addition, questions DC009–DC018 are used in combination with the depression level scale to quantify the data of mental health rating as a main variable to measure depression status in mental health to comprehensively reflect the degree of depression among the rural older adult.

This study references the research methods of Tao and Shen (26) regarding indicators of mental health. Through factor analysis, three principal components are extracted which represent first-level indicators. The proportion of each rotating squared sum load to the total load of the three principal components is obtained via maximum variance rotation, to setting the weights of these three dimensions to 34.3116%, 33.1109%, and 32.5775%. Since the proportions of the three dimensions of mental health in the 2018 CHALRS questionnaire are equally important, this study directly applies one-third weight to the secondary indicators of mental health. Applying the entropy weight method to the three indicators to ensure rationality and accuracy, the objective assignment comprehensively considers errors and distortions, finally determining the entropy weight of the mental health dimension according to the distribution method presented in Table 2, which divides the mental health dimension in more detail.

**Table 2:** Mental health right weight table.

First grade indexes	Second index	Weight	Third grade indexes	Weight
Mental health	Life self-evaluation satisfaction	1/3	Overall, are you satisfied with your life	0.3333
			MMS Memory 1 (10 questions)	0.0192
			MMS Memory 2 (10 questions)	0.0152
			MMS Memory 3 (10 questions)	0.015
	Cognitive scale	1/3	MMS Directional force 1	0.0557
			MMS Directional force 2	0.0047
			MMS Directional force 3	0.0513
			MMS Basic cognition 1	0.0539
			MMS Basic cognition 2	0.0560
			MMS Basic cognition 3	0.0156
			MMS Basic cognition 4	0.0467
	Depression scale	1/3	I’m troubled by some small things	0.0323
			It is difficult for me to concentrate when doing things.	0.0297
			I’m feeling down.	0.0293
			I find it hard to do anything.	0.0278
			I am full of hope for the future	0.0214
			I feel afraid	0.0579
			My sleep is not good	0.0278
			I am very happy	0.0140
			I feel lonely.	0.0436
			I feel like I can’t go on with my life	0.0496

##### Physical health ( psh )

In this study, residents’ daily living self-care ability (ADL) represents rural older adult residents’ objective physiological health status. Seven indicators of ADL are used, including whether respondents can run or jog for 1 km, bend and bend their knees or squat, dress independently, bathe independently, eat independently, go to the toilet independently and control physiological excretion. According to the internationally accepted definition, if one or more of the above seven activities of daily living require assistance from others to complete, respondents are considered to have daily living ability limitations, and the ADL health index is assigned to 0. Conversely, if the seven activities of daily living can be completed independently, the ADL health index is assigned to 1. The specific assignments are presented in Table 1.

##### Control variables

Referencing Grossman’s health demand theory (25) and Liu and Liu (43), this study uses three groups of control variables. The first is individual characteristics, including age ( age ), sex ( sex ) and marital status ( mst ). The second includes the economic characteristic of income level ( icl ), and the third is social characteristics, covering social stratum ( sst ).

##### Mediating variables

To further explore the impact of the NRSPI on the physical and mental health of the rural older adult, this study references Wu et al. (52), introducing mediating variables of social, life and spiritual levels and setting medical services ( msv ), food acquisition ( fas ) and recreational activities ( rac ) as mediating variables to examine how the NRSPI affects health status based on these variables.

### Data sources

This study takes the rural older adult in China as the research sample, and micro-data employed are from the 2015 and 2018 CHARLS conducted by the National Development Research Institute of Peking University and implemented by the China Social Science Survey Centre. The main reason for choosing this database is its alignment with international experience, including the United States Health and Retirement Survey, the British Longitudinal Ageing Survey and the European Health, Ageing and Retirement Survey (SHARE) and pioneering electronic mapping software (CHARLS-GIS) technology, using a mapping method which produces village-level sampling frames. The samples cover more than 150 county-level units and 450 community-(village) level units in 28 provinces (autonomous regions and municipalities) across China, representing 19,000 respondents and 12,400 households. These data are high-quality and provide micro-level information on households and individuals of middle-age and older adult people aged 45 and above. In order to obtain the net effect of NRSPI, this study separates NRSPI from other types of pension or pension insurance, and further screens the samples, excluding individuals who had participated in the Old rural social pension insurance before 2009 and had retired and enjoyed pension or pension in 2009. In addition, in order to study the impact of NRSPI on the health status of the rural older adult, this paper only selects the rural older adult aged 60 and over in the sample, and the age distribution between the samples is relatively concentrated. The effect of age segmentation on the regression results is not significant, so the urban–rural distribution and age segmentation are eliminated from the individual characteristics. The final analysis data contains 1,660 samples. At the same time, according to the needs of the robustness test part, this paper synthesizes the CHARLS 2015 and 2018 survey data into panel data for the PSM-DID test.

### Descriptive statistics

Table 1 presents the descriptive statistical analysis results of the main variables. The explanatory variable used examines whether the respondent participated in the NRSPI, with an average of 0.761. The purchase rate, implementation and participation effect of the NRSPI can be directly quantified using the CHARLS (2018) survey data. The explained variables include self-reported health evaluation, mental health and physical health, which are all ordered categorical variables with values of 1–5, and the mean values are 3.092, 3.587 and 3.255, respectively.

## Empirical results and additional tests

### Benchmark regression results

This study adopts a step-wise regression analysis method. Columns (2) and (3) of Table 3 present the regression results using self-reported health evaluation as the explained variable, columns (4) and (5) present the regression results using mental health as the explained variable and columns (6) and (7) present the regression results using physical health as the explained variable. Columns (2) and (6) of Table 3 reveal that the NRSPI has a significant positive impact on the self-reported health evaluation and physical health of the rural older adult at the 1% level. The rural older adult who benefit from the NRSPI exhibit improved self-reported health evaluation and physical health compared with those who do not participate in the NRSPI. The impact coefficient of the NRSPI on the self-reported health evaluation and physical health of the rural older adult decreases after introducing control variables but remains significantly positive. This demonstrates that the NRSPI improves the economic circumstances of the rural older adult and increases the variety of food acquisition and medical service expenditure, which improves physical health. Individuals in similar physical condition with more income channels have sufficient social support and more optimistic self-reported health evaluation due to higher overall income, which has a positive impact on self-reported health evaluation and physical health (3, 8, 26). Notably, the regression results show that although the influence coefficient of the NRSPI on mental health is positive, it is not significant, indicating that the NRSPI exerts a minimal positive impact on the mental health of the rural older adult. The relatively stable pension can mitigate financial worries and increase leisure and entertainment activities to elicit optimistic attitudes and alleviate the depression of the rural older adult. It also reduces the inter-generational support and spiritual comfort of children, which is cited as the primary reason the mental health problems of the insured rural older adult remain prominent. The regression results also verify the conclusions of the existing literature (43–45). Hypothesis 1 is verified.

**Table 3:** Logistic regression results.

Variable name	Model 1	Model 2	Model 3	Model 4	Model 5	Model 6
	sfe	sfe	mth	mth	psh	psh
nrspi	0.126*** (3.17)	0.017*** (3.21)	0.149 (1.44)	0.004 (0.06)	0.119*** (4.13)	0.012** (2.07)
age		−0.024** (−2.23)		−0.043 (−0.04)		−0.027***(−3.92)
sex		0.326** (2.42)		0.217** (2.31)		0.362*** (3.98)
mst		0.122 (1.14)		0.096** (2.36)		0.117 (1.19)
icl		0.118*** (3.42)		0.144*** (3.16)		0.209*** (4.07)
sst		0.091*** (3.26)		0.116*** (3.98)		0.094*** (3.37)
*N*	1,660	1,660	1,660	1,660	1,660	1,660
Pseudo R2	0.0108	0.0367	0.0112	0.0574	0.0116	0.0397

*, **, *** are significant at the levels of 10%, 5%, and 1%, respectively, and the corresponding *t* values are in parentheses.

### Heterogeneity analyses

Influenced by historical factors, traditional concepts and physical characteristics, considerable differences are evident in the social and economic status of rural older adult groups of different genders, which affect health status. This study examines the impact of the NRSPI on the physical and mental health of rural older adult men and women. The regression results in Table 4 indicate that the impact of the NRSPI on the health of the rural older adult of different genders is simultaneously consistent and diverse. The NRSPI has a significant impact on the self-reported health evaluation of older adult rural males and females at the 1% level, revealing that older adult males and females who participate in the NRSPI have higher self-reported health evaluation than those who do not participate. In addition, the NRSPI has a significant impact on the physical health of rural older adult females, but the impact on males is not significant. The rationale for this difference may be the considerable differences in the social economy between older adult males and females in rural areas and notable differences in the timeliness and feasibility of older adult care and medical treatment for older adult males and females. The above heterogeneity analysis results also prove the reliability of the previous analysis (27, 32). In addition, as with previous findings, the impact of the NRSPI on the mental health of rural older adult men and women is not significant.

**Table 4:** Heterogeneity regression results.

Variable name	Male older adult			Female older adult		
	Model 7a	Model 7b	Model 7c	Model 8a	Model 8b	Model 8c
	sfe	mth	psh	sfe	mth	psh
nrspi	0.059*** (3.46)	0.097 (0.62)	0.154 (0.93)	0.015*** (3.12)	0.098 (0.67)	0.056*** (3.34)
age	−0.025*** (−3.61)	−0.006 (−0.68)	−0.018** (−2.21)	−0.017** (−2.12)	0.037** (2.43)	0.037*** (3.64)
mst	−0.237 (−1.44)	−0.078 (0.41)	−0.336** (−2.03)	0.037* (1.76)	0.094*** (4.63)	0.013 (0.17)
icl	0.542*** (3.97)	0.106*** (3.63)	0.157*** (4.54)	0.244*** (2.73)	0.278*** (2.61)	0.343*** (3.68)
sst	0.126*** (2.94)	0.122*** (2.78)	0.096*** (3.14)	0.068*** (3.67)	0.124*** (2.84)	0.113*** (2.76)
*N*	800	800	800	860	860	860
Pseudo R2	0.0423	0.0567	0.0368	0.0304	0.0565	0.0391

*, **, *** are significant at the levels of 10%, 5%, and 1%, respectively, and the corresponding *t* values are in parentheses.

### Mediating effect test

To explore whether the NRSPI can promote the health status of the rural older adult population through mediating variables, this paper empirically tests the role of medical services, food acquisition and recreational activities applying a step-wise regression method. Column (2) of Tables 5–7 presents the respective regression results for medical services, food acquisition and recreational activities as mediating variables in Eq. 4. The results reveal that the coefficients of the NRSPI are significantly positive at the 1% level, indicating that medical services, food acquisition and recreational activities have mediating effects on the NRSPI.

**Table 5:** The mediating effect test of medical services.

Variable name	Model 4	Model 5	Model 6	Model 7
	msv	sfe	mth	psh
nrspi	0.227*** (3.19)	0.014*** (3.17)	0.011 (1.06)	0.008** (2.13)
msv		0.089** (2.14)	0.757*** (3.56)	0.365** (2.43)
age	−0.032* (−1.73)	−0.022** (−2.21)	−0.143 (−0.16)	−0.019*** (−3.74)
sex	0.169*** (3.62)	0.372** (2.16)	0.231** (2.42)	0.372*** (2.97)
mst	0.132 (1.43)	0.171 (1.19)	0.092** (2.36)	0.126 (1.05)
icl	0.162*** (2.83)	0.117*** (3.53)	0.146*** (2.97)	0.201*** (4.12)
sst	0.167*** (2.68)	0.093*** (4.19)	0.119*** (3.94)	0.097*** (3.32)
*N*	1,660	1,660	1,660	1,660
Pseudo R2	0.0147	0.0428	0.0602	0.0413

*, **, *** are significant at the levels of 10%, 5%, and 1%, respectively, and the corresponding *t* values are in parentheses.

**Table 6:** The mediating effect test of food acquisition.

Variable name	Model 4	Model 5	Model 6	Model 7
	fas	sfe	mth	psh
nrspi	0.427*** (3.42)	0.015*** (3.05)	0.003 (0.05)	0.010** (2.05)
fas		0.017** (2.06)	0.004** (2.13)	0.174*** (3.91)
age	−0.026 (−0.12)	−0.031 (−0.69)	−0.043 (−1.13)	−0.036*** (−3.64)
sex	0.401** (2.33)	0.317** (2.12)	0.382** (2.41)	0.268*** (2.85)
mst	0.069** (2.14)	0.073** (2.17)	0.046** (2.48)	0.132 (1.49)
icl	0.127*** (3.13)	0.133*** (2.82)	0.188*** (2.69)	0.214*** (3.94)
sst	0.182*** (3.56)	0.134*** (3.17)	0.163*** (3.64)	0.089*** (3.89)
*N*	1,660	1,660	1,660	1,660
Pseudo R2	0.0153	0.0504	0.0618	0.0407

*, **, *** are significant at the levels of 10%, 5%, and 1%, respectively, and the corresponding *t* values are in parentheses.

**Table 7:** Test of the mediating effect of entertainment activities.

Variable name	Model 4	Model 5	Model 6	Model 7
	rac	sfe	mth	psh
nrspi	0.531*** (4.02)	0.008** (2.09)	0.001 (0.17)	0.013*** (3.64)
rac		0.211*** (2.83)	0.136** (2.49)	0.053*** (4.17)
age	−0.031*** (−3.42)	−0.016*** (−3.73)	−0.049 (−0.78)	−0.027*** (−3.44)
sex	0.273*** (3.87)	0.326*** (3.46)	0.253** (2.38)	0.349*** (3.98)
mst	0.121 (1.32)	0.104 (1.13)	0.078** (2.41)	0.128 (1.17)
icl	0.236*** (4.07)	0.283*** (3.92)	0.152*** (2.87)	0.214*** (3.73)
sst	0.112*** (3.21)	0.081*** (2.87)	0.103*** (3.46)	0.069*** (3.74)
*N*	1,660	1,660	1,660	1,660
Pseudo R2	0.0141	0.0473	0.0602	0.0414

*, **, *** are significant at the levels of 10%, 5%, and 1%, respectively, and the corresponding *t* values are in parentheses.

This paper further regresses the Eqs 5–7, presenting the results in columns (2–4) of Tables 5–7. After introducing the three mediating variables, the coefficient of the NRSPI on self-reported health evaluation and physical health remain significantly positive, and the coefficient of mental health is positive but not significant. The regression results demonstrate that the NRSPI has improved rural older adult population health by strengthening medical services, enriching the types of food acquisition and increasing the amount of leisure and recreational activities enjoyed. A possible rationale is that the NRSPI provides stable pension income for the insured rural older adult and encourages them to use this income to increase the variety of food intake and medical services in daily life, which improves dietary balance and nutritional intake, reducing the probability of illness, improving self-care ability and promoting the improvement of physical health. In addition, pension income also reduces the labour participation time of the insured older adult, increases participation in social recreational activities, reduces the concerns about old age, improves life satisfaction and improves mental health, resulting in more optimistic self-reported health evaluations. Hypotheses 2 and 3 are confirmed.

### Moderating effect test

Older adult health is a complex and evolving challenge. Compared with the urban older adult, the health risks of the rural older adult are affected by multiple factors with mutual influences between the related dimensions. For example, the older adult with good mental health are capable of improving physical health, while those with poor physical health are more likely to have low psychological dispositions or pessimistic attitudes towards life. Therefore, when the health status of the older adult in a single dimension is problematic, the probability that in other dimensions falling into risk will inevitably increase. To control the NRSPI, control variables and mediating variables, this study empirically tests the moderating effect of the NRSPI on the physical and mental health of the rural older adult as shown in Table 8.

**Table 8:** Regulating effect test.

Variable name	Model 9a	Model 9b	Model 9c	Model 10a	Model 10b	Model 10c
	mth	mth	mth	psh	psh	psh
nrspi	Controlled	Controlled	Controlled	Controlled	Controlled	Controlled
Control variable		Controlled	Controlled		Controlled	Controlled
Mediator variable			Controlled			Controlled
mth				1.113*** (3.64)	1.062*** (3.56)	1.006*** (3.37)
psh	1.238*** (2.83)	1.216*** (2.94)	1.157*** (2.62)			
mth×psh	3.086*** (2.84)	3.094*** (2.97)	3.103*** (2.59)	3.526*** (3.03)	3.504*** (2.92)	3.481*** (2.87)
*N*	1,660	1,660	1,660	1,660	1,660	1,660
Pseudo R2	0.8452	0.8469	0.8487	0.9034	0.9041	0.9058

*, **, *** are significant at the levels of 10%, 5%, and 1%, respectively, and the corresponding *t* values are in parentheses.

The results of the regression model indicate that the physical health of the rural older adult has a significant positive effect on mental health, which was verified in the three regression models. In the model progressively introducing the NRSPI, control variables and mediating variables, the correlation of mental health with physical health for the older adult in rural areas is 1.237 times, 1.219 times and 1.159 times that of the older adult with physical health challenges, and the NRSPI has a 7.8% moderating effect. In addition, the mental health of the rural older adult also exhibits a positive impact on physical health. In the full variable model, the correlation of physical health challenges with mental health is 1.004 times that of the rural older adult with mental health challenges, demonstrating that mental health has a certain transformative effect on physical health, where the NRSPI has a 10.7% adjustment effect.

### Robustness test

In this study, the use of multivariate ordinal logistic regression can largely control the endogenous bias caused by observable and unobservable variables, but it cannot effectively avoid the estimation bias caused by heterogeneity over time. Therefore, robustness tests are conducted to further confirm the reliability of the research results. First, before performing the difference-in-differences estimation (PSM-DID), it is usually necessary to perform a balanced trend test. The test methods include t-test method, graphical method, interaction term regression method and F-statistic method. In this paper, the t test method is used. Table 9 shows the parallel trend test of *t* test method, that is, when dealing with the data before the experiment, the processing variables and the research variables are tested. Because there is no significant difference, it shows that the parallel trend test is satisfied. At the same time, Table 9 also shows the results of the *t*-test between the post-processing variables and the explained variables. The t value shows a significant difference, indicating that there is a significant difference between the control group and the experimental group after the experiment. Second, this study uses the PSM-DID model to test the health impact of the NRSPI on the rural older adult by using the panel data from different years. The CHARLS 2015 and 2018 survey data are used to reconstruct the panel data for robustness testing. If the impact of NRSPI policy on the health of the rural older adult remains valid, this indicates that the benchmark empirical results are robust. Table 10 reveals that among the five models, the DID interaction coefficient and the regression coefficients of the three mediating variables remain significant, confirming that the benchmark results regarding the impact of NRSPI on the health of the rural older adult and the impact mechanisms are robust. In addition, this study uses the diff command in STATA software to test the previous results, revealing that the regression coefficient of the NRSPI on the health of the rural older adult remains significant at the 10% level, once again verifying that the benchmark results are robust.

**Table 9:** *T* test.

Item	Before				After			
	Control	Treated	Diff	*t*	Control	Treated	Diff	*t*
sfe	0.021	0.032	0.011	1.06	0.018	0.041	0.023	3.43
mth	0.008	0.012	0.004	1.12	0.003	0.009	0.006	1.98
psh	0.028	0.036	0.008	1.33	0.022	0.046	0.024	2.11
Control variable	Controlled	Controlled	Controlled	Controlled	Controlled	Controlled	Controlled	Controlled

**Table 10:** Robustness test.

Variable name	Model 1	Model 2	Model 3	Model 4	Model 5
Treatment group × Year	0.032* (1.78)	0.028* (1.92)	0.027** (2.32)	0.026* (1.87)	0.024* (1.77)
Year	0.132*** (4.66)	−0.116*** (3.26)	0.083*** (3.05)	0.067*** (3.54)	0.065*** (2.98)
msv			0.041*** (3.43)	0.046*** (4.21)	0.043*** (2.87)
rac				0.014*** (2.98)	0.016*** (3.12)
fas					0.008* (1.84)
Control variable	Uncontrolled	Controlled	Controlled	Controlled	Controlled
R2	0.05	0.07	0.14	0.17	0.17

*, **, *** are significant at the levels of 10%, 5%, and 1%, respectively, and the corresponding *t* values are in parentheses.

## Conclusions and recommendations

Based on 2015 and 2018 CHARLS survey data, this study applies the multivariate ordered logistic regression model to empirically analyse the impact of the NRSPI on the health of the rural older adult population, drawing four relevant conclusions.

1. The benchmark regression results demonstrate that the NRSPI has a positive impact on the health of China’s rural older adult. The NRSPI is significant at the 1% level and has a positive impact on the self-reported and physical health of the rural older adult. The impact of NRSPI on the mental health of the rural older adult is not significant but the coefficient is still positive.
2. The heterogeneity analysis results show that the NRSPI has a significant impact on the self-reported health of rural male and female older adult at the 1% level. In addition, the NRSPI has a significant impact on the physical health of rural female older adult respondents, while the impact on males is not significant. In addition, the impact of the NRSPI on the mental health of rural male and female older adult is not significant.
3. The mediating effect test results reveal that medical services, food access and entertainment activities have a mediating effect on the NRSPI. After introducing the three mediating variables, the coefficients of self-reported and physical health and the NRSPI remain significantly positive, and that of mental health is not significant but still positive.
4. The moderating effect test results indicate that the mental health and physical health of the rural older adult have a certain transformative effect. In the model progressively introducing the NRSPI, control variables and mediating variables, the NRSPI adjusted 7.8% of the effect of physical health on mental health and 10.7% of the effect of mental health on physical health.

Under the background of Healthy China strategy implementation, this study proposes the following three suggestions to advance the system of healthy old-age care for the rural older adult population based on the empirical results.

1. Improve the coverage of the NRSPI. Although the NRSPI has achieved the goal of wide coverage, the implementation of the family-bundled insurance policy and personal payments for the NRSPI increasing annually results in a considerable number of rural older adult people being omitted by the system, some of whom have even given up and are unable to obtain the protection of healthy old-age care. Accordingly, the government should gradually phase out the family bundling policy and reasonably optimise the personal payments required by the NRSPI. A social pension service system with government support and diversified development should be strategically targeted in relation to the unique economic incomes and personal pension needs of the rural older adult, continuing to expand the coverage of the NRSPI and solidly promoting the full participation of the rural older adult population.
2. Moderately improve the level of NRSPI benefits and improve the dynamic adjustment system. China established the NRSPI system to cover the whole country, which has an important role in ensuring the lives and health of the older adult in rural areas. However, the basic pension of the NRSPI implemented in 2009 was 55 yuan per month, while the basic pension of the new rural insurance after its merger in 2015 was only raised to 70 yuan per month. Overall, the financial support provided by the NRSPI remains relatively low, restricting the potential effects of the NRSPI system on improving the lives and health of the rural older adult. Consequently, the government should continue to improve the dynamic adjustment mechanism of the NRSPI on the basis of government expenditure, establish a rational growth mechanism between the basic old-age insurance fund and the consumer index, improve the level of pension treatment and gradually narrow the gap between the treatment of urban and rural residents so that NRSPI can effectively protect the basic lives and health of the rural older adult. The positive role of the NRSPI will further expand the beneficiaries of this new insurance programme and increase the benefits for the rural older adult.
3. Prioritise guiding the rural older adult to participate in the NRSPI and strengthen the publicity and guidance towards healthy lifestyles. By increasing social activities such as medical services, leisure and entertainment and improving the variety of food access and dietary balance, public leaders can improve rural older adult population health and enrich and improve the rural older adult’s sense of belonging and achievement to maintain their positive and optimistic attitudes and health status and maximise the role of the NRSPI in advancing healthy lifestyles among the rural older adult.
4. Initiate the development of emotional support systems in rural old-age services. With the gradual improvement of China’s socialised old-age care system, social subjects can be stimulated to support and care for the older adult in rural areas through publicity and education, establishing a social atmosphere that respects and helps the older adult in rural areas and mitigates loneliness, tension and a lack of belonging, forming a society that values and cares for the older adult in rural areas, improving their well-being and sense of security and improving their physical and mental health.

## Data availability statement

The original contributions presented in the study are included in the article/supplementary material, further inquiries can be directed to the corresponding author.

## Author contributions

YS: Conceptualization, Formal analysis, Funding acquisition, Writing – original draft. CS: Data curation, Writing – original draft, Formal analysis. ZW: Conceptualization, Data curation, Writing – original draft. GH: Conceptualization, Project administration, Writing – original draft.
